# Translational Research Opportunities Regarding Homologous Recombination in Ovarian Cancer

**DOI:** 10.3390/ijms19103249

**Published:** 2018-10-19

**Authors:** Margarita Romeo, Juan Carlos Pardo, Anna Martínez-Cardús, Eva Martínez-Balibrea, Vanesa Quiroga, Sergio Martínez-Román, Francesc Solé, Mireia Margelí, Ricard Mesía

**Affiliations:** 1 Medical Oncology Department, B-ARGO Group, Institut Català d’Oncologia Badalona, Carretera del Canyet s/n, 08916 Badalona, Spain; jcpardor@iconcologia.net (J.C.P.); vquiroga@iconcologia.net (V.Q.); mmargeli@iconcologia.net (M.M.); rmesia@iconcologia.net (R.M.); 2 Campus de la UAB, Universitat Autónoma de Barcelona, Plaça Cívica, 08193 Bellaterra, Spain; 3 Health Sciences Research Institute of the Germans Trias i Pujol Foundation (IGTP), B-ARGO Group, Carretera del Canyet s/n, 08916 Badalona, Spain; amartinezc@igtp.cat; 4 Program against Cancer Therapeutic Resistance (ProCURE), Institut Català d’Oncologia Badalona, Program for Predictive and Personalized Cancer Medicine (PMPPC), Health Sciences Research Institute Germans Trias i Pujo (IGTP), Carretera de Can Ruti, Camí de les Escoles s/n, 08916 Badalona, Spain; embalibrea@iconcologia.net; 5 Gynecology Department, Hospital Universitari Germans Trias i Pujol, 08916 Badalona, Spain; smartinezro.germanstrias@gencat.cat; 6 Institut de Recerca contra la Leucemia Josep Carreras, 08916 Badalona, Spain; fsole@carrerasresearch.org

**Keywords:** ovarian cancer, high-grade serous ovarian cancer, deficient homologous recombination, PARP inhibitors, BRCA1, BRCA2, mechanisms of resistance

## Abstract

Homologous recombination (HR) is a DNA repair pathway that is deficient in 50% of high-grade serous ovarian carcinomas (HGSOC). Deficient HR (DHR) constitutes a therapeutic opportunity for these patients, thanks to poly (ADP-ribose) polymerases (PARP) inhibitors (PARPi; olaparib, niraparib, and rucaparib are already commercialized). Although initially, PARPi were developed for patients with *BRCA*1/2 mutations, robust clinical data have shown their benefit in a broader population without DHR. This breakthrough in daily practice has raised several questions that necessitate further research: How can populations that will most benefit from PARPi be selected? At which stage of ovarian cancer should PARPi be used? Which strategies are reasonable to overcome PARPi resistance? In this paper, we present a summary of the literature and discuss the present clinical research involving PARPi (after reviewing ClinicalTrials.gov) from a translational perspective. Research into the functional biomarkers of DHR and clinical trials testing PARPi benefits as first-line setting or rechallenge are currently ongoing. Additionally, in the clinical setting, only secondary restoring mutations of *BRCA*1/2 have been identified as events inducing resistance to PARPi. The clinical frequency of this and other mechanisms that have been described in preclinics is unknown. It is of great importance to study mechanisms of resistance to PARPi to guide the clinical development of drug combinations.

## 1. Introduction

Homologous recombination (HR) is an error-free DNA-repair system that is activated in cases of double-strand damage, such as that induced by ultraviolet light, spontaneous mutations, and some chemotherapies (e.g., platinum salts) [1]. This pathway acts by building a homology-directed copy of the sister chromatid during the S and G2 phases. *Breast cancer gene* 1 (*BRCA*1) and, to a lesser extent, *breast cancer gene* 2 (*BRCA*2) play key roles in HR [2]. DNA double-strand breaks (DSBs) can cause cellular apoptosis if not repaired in time, but *BRCA1*, in particular, coordinates the repair response by recruiting several proteins, and ultimately contributes to the maintenance of the genome integrity and cell survival. In contrast, BRCA2 has a very precise role in the HR by interacting with RAD51 recombinase (RAD51). RAD51 is involved in a very significant step of HR; it is the recombinase that promotes the invasion of the preserved sister chromatid that serves as a mold to rebuild a copy with high fidelity. During the S/G2 phases of the cell cycle, RAD51 accumulates at DSBs and forms microscopically visible subnuclear foci [3].

*BRCA*1/2 deficiency causes HR impairment and is associated with breast and ovarian carcinogenesis [2]. Their pathologic germline mutations were described long time ago to be linked to the hereditary syndrome of breast and ovarian cancers. In fact, they are present in 50% of hereditary high-grade serous ovarian cancers (HGSOC), which is the most frequent pathologic subtype. Truncating somatic mutations affect an additional small proportion of sporadic HGSOCs (<7%) [4]. However, the most frequent event causing *BRCA*1 inactivation in sporadic HGSOC is its promoter hypermethylation (~15%) [5]. Loss of heterozygosity (LOH) usually occurs when one allele of *BRCA*1/2 harbours pathologic mutations, leading to total *BRCA*1/2 inactivation and very low or undetectable BRCA1/2 expression [6]. In fact, The Genome Cancer Atlas project in ovarian cancer (TGCA-Ov), which is focused on HGSOC, showed that this particular subtype is characterized by high genomic instability and tumor protein p53 (p53) functional loss in all cases, as well as deficient HR (DHR) in 50% of cases. This study also identified other defects related to DHR beyond *BRCA*1/2 alterations, such as mutations or methylations of *ATM*, *BARD1*, *BRIP1*, *CHEK1*, *CHEK2*, *FANCL*, *PALB2*, *PPP2R2A*, *RAD51B*, *RAD51C*, *RAD51D*, *RAD54L*, *ATR*, *BARD1*, *NBN*, *RAD50*, *FAM175A*, and *MRE11A* [4].

HGSOCs associated with germinal *BRCA*1/2 mutations have some common clinical features that are included under the term “BRCAness phenotype” (or absence of functional BRCA phenotype). Numerous retrospective studies have described more frequent visceral metastases at the debut of disease [7]. Strong evidence indicates that *BRCA*1/2 mutations are associated with improved survival in HGSOC patients after adjustment for staging [7]. In 2012, Bolton et al. [8] published a pooled analysis of 1213 patients from 36 different studies, showing five-year overall survival rates (OS) of 36%, 44% and 52% for non-mutated patients, patients with the *BRCA*1 mutation and patients with the germinal *BRCA*2 mutation, respectively. There was a statistically significant survival benefit for patients with a mutation in either gene relative to non-mutated genes [g*BRCA*1 mut: hazard ratio (hr) 0.78, 95% CI 0.68–0.89; g*BRCA*2 mut: hr 0.61, 95% CI 0.50–0.76]. While the best prognosis of these tumors is hypothesized to be related to increased platinum sensitivity, it cannot be ruled out that they present different natural histories related to greater lymphocyte infiltration [7]. Moreover, the published phase I trial of olaparib written by Fong et al. pointed at *BRCA*1/2 mutated cancers as good candidates for poly (ADP-ribose) polymerases (PARP) inhibitors (PARPi) treatment and attributed the antitumor activity of these molecules to an effect called synthetic lethality [9].

The family of PARPs catalyzes the addition of polyAPD-ribose groups from the NAD+ dinucleotide to phosphate groups of certain proteins, modifying their cellular function (PARylation). PARP1 is particularly involved in DNA-repair mechanisms. PARP1 accumulates in single-strand DNA breaks, contributing to the recruitment of several proteins involved in base-excision repair (BER), and regulating transcription through histone PARylation. Upon completion of these tasks, autorybosilation of PARP1 allows its dissociation from DNA [10]. PARPi compete with NAD+, thus inhibiting PARP catalytic activity, and causing the trapping of PARP molecules (PARP trapping) in DNA damage points. This latter fact provokes a stop in the replication forks and can induce increased apoptosis than inhibition of PARP catalytic activity [10,11]. On the whole, PARP inhibition induces the accumulation of single-strand DNA damage, which, in turn, can result in DSBs. Cells with inactive HR are not able to repair these DSBs, causing the cell to undergo apoptosis. In the case of HGSOCs with *BRCA*1/2 mutations, this effect is cytotoxic for tumor cells. This mechanism of cell death mediated by the simultaneous failure of two DNA repair mechanisms has been called “synthetic lethality” [12]. This was the initial basis for the development of PARPi. There are alternative or complementary hypotheses that aim to explain the mechanism of action of PARPi related to the role of PARP in the regulation of HR, non homologous end joining (NHEJ), and alternative end joining (A-EJ) [13]. However, these are only partially understood. Nowadays, although PARPi have proved to be useful in a broader population than exclusively *BRCA*1/2-mutated patients, these alterations are the strongest predictive factor of response to PARPi. In addition, since the beginning of the clinical development of PARPi in the late 2000s, they have obtained several approvals in ovarian cancer from drug regulatory agencies. Future approvals for breast, pancreatic and prostate cancers are expected.

There are several PARPi in development, but only three have been already commercialized: olaparib (O, first-in-class), niraparib (N), and rucaparib (R). O and R inhibit PARP1, PARP2 and PARP3, while N only inhibits PARP1 and PARP2. The three molecules inhibit catalytic PARP1 activity with different levels of potency (IC50 values: O, 1.2 nmol/L; N, 50.5 nmol/L; R, 21 nmol/L) and different capabilities to trap PARP1 in the replication forks (greater for N) [11]. Clinically, the first trials with O showed high response rates (at a dose of 400 mg daily) in highly pretreated patients, between 24% and 40% of patients with *BRCA*1/2-mutated associated triple-negative breast cancer or HGSOC [9,14,15]. Updated approvals by the European Medicines Agency (EMA) and the US Food and Drug Administration (FDA) are summarized in Table 1. O was first approved by FDA as a treatment for relapsed HGSOC associated with germline *BRCA*1/2 mutations after progression to three or more previous chemotherapy lines [16]. This approval is based on a phase II trial with 193 platinum-resistant relapsed patients (or not candidates to retreatment with platinum salts), in which investigators observed a rate of objective responses of 34% (95% CI: 26–42) and an OS of 16.6 months [17]. In contrast, in Europe, O was first approved for patients with *BRCA*1/2 mutated-associated HGSOC as a maintenance treatment following response to platinum salts used for recurrence [18]. This indication was based on the Nineteen study, a phase II trial that showed an absolute benefit in the progression-free survival (PFS) of seven months (hr: 0.18, *p* < 0.0001) in the subgroup of patients with *BRCA*1/2 mutations (retrospective preplanned subgroup analyses, *n* = 136) [19,20,21]. Recent publication of the results of the phase III trial SOLO2, including only *BRCA*1/2-mutated patients, supports this approval, showing an absolute benefit of nearly 14 months (hr: 0.30, *p* < 0.0001) [22]. Recently, FDA granted O with the maintenance indication without molecular selection, based on data from the Nineteen study showing hr of 0.35, *p* < 0.001, in the intention-to-treat analyses including patients with or without *BRCA*1/2 mutations following response to platinum-based chemotherapy used for relapse treatment (*n* = 265). Moreover, EMA has recently given a post-authorization positive opinion on this indication [19]. Confirmatory results from two phase III-IV trials are expected (see below). In addition, N was approved in Europe and US in the maintenance setting for “all comers” (without molecular selection) [18] based on the NOVA trial. Its results indicate an absolute PFS benefit of five months in *BRCA*1/2 wild-type patients (hr: 0.45, *p* < 0.001), nine months in *BRCA*1/2 wild-type patients with DHR (hr: 0.38, *p* < 0.001), and sixteen months in *BRCA*1/2-mutated patients (hr: 0.27, *p* < 0.001) [23]. In 2018, R has also obtained FDA approval for this same indication, based on results obtained in the ARIEL3 randomized placebo-controlled trial [24]. However, in contrast, its first indication was obtained from the FDA as a monotherapy for relapses or progression after two or more lines of chemotherapy in patients with *BRCA*1/2 mutations who are unable to tolerate further platinum-based chemotherapy. This was based on two phase II studies whose global analyses showed a response rate of 54% (9% complete) with a median duration of 9 months [16,25,26]. In May 2018, R has obtained a similar indication from EMA restricted to patients with platinum-sensitive relapse unable to tolerate further platinum-based chemotherapy. Regarding the toxicity reported in the three maintenance studies (Nineteen, NOVA and ARIEL3), the most frequent non-hematological grade 3 adverse events were nausea/emesis and fatigue, which occurred in 2 to 4% and 6 to 9% of cases, respectively. Hematological toxicity is also relevant, but its profile differs among the three drugs: N alters the three series (20 to 34% of patients with grade 3–4 events), while O and R cause anemia, in particular (17% and 22% grade 3–4 events, respectively) [19,23,24].

In summary, HR is a DNA-repair pathway that is frequently deficient in HGSOC. This constitutes a therapeutic opportunity for these patients, thanks to PARPi. Although initially these drugs were developed for patients with *BRCA*1/2 mutations, robust clinical data showing their benefit in a broader population without DHR are now available. This breakthrough in daily practice raises many other unanswered questions that represent opportunities for translational research, such as (1) the selection of the population that will most benefit from such treatments; (2) the stage of disease that they should be used; and (3) the formation of strategies overcome resistance to PARPi. Our goal is to discuss each of these topics from a translational perspective.

## 2. Open Questions

### 2.1. Choicing Good Candidates for PARPi

The BRCAness phenotype has been attributed to DHR and it could potentially be extrapolated to other patients with HR defects other than germinal *BRCA*1/2 mutations. As stated before, PARPi were initially developed for germline *BRCA*-mutated patients under the synthetic lethality hypothesis [27]. In this section, we will summarize which molecular tumor features may indicate sensitivity to PARPi (Reviewed in Hoppe 2018 [28]).

#### 2.1.1. Somatic *BRCA*1/2 Mutations

Subsequent published research has suggested a similar prognosis between germline and somatic *BRCA*1/2 mutations. Pennington showed that somatic *BRCA*1/2 mutations have similar positive impacts on OS and platinum responsiveness as germline *BRCA*1/2 mutations [19]. Although clinical trials suggest that somatic and germline mutations have similar predictive roles in the response to PARPi (ARIEL2 and ARIEL3 trials, Nineteen, NOVA), the body of evidence is small due to the small proportion of somatic *BRCA*1/2 mutations. Specifically, the NOVA trial performed an exploratory analysis with 47 patients that harbored somatic mutations in *BRCA*1/2 and found that the benefit of N was identical to that found in patients with germline mutations [hr: 0.27 (95% CI: 0.08–0.90); and hr: 0.27 (95% CI: 0.17–41), respectively] [23]. A current trial involving the use of O as a maintenance drug after response to retreatment with platinum aims to recruit 54 patients with somatic *BRCA*1/2-mutated tumors (ORZORA trial, NCT02476968) [29]. In addition, the impacts of specific *BRCA*1 or *BRCA*2 mutations or the absence of *BRCA* locus-specific LOH on the prognosis and response to PARPi are still unknown [24,28,30,31].

#### 2.1.2. BRCA1 Promoter Hypermethylation

On the other hand, there is discordant literature regarding the impact of *BRCA*1-promoter hypermethylation on HGSOC prognosis. A few retrospective clinical studies have suggested that low expression of *BRCA*1, measured either by RNA quantification or by immunochemistry, may be associated with greater sensitivity to platinum compounds [32,33]. However, the TGCA-Ov study (where 94% of the patients had received a combination of platinum with taxanes) provided evidence in favor of different prognosis between tumors with mutations of *BRCA*1/2 and those with *BRCA*1-promoter hypermethylation (similar to *BRCA*1/2 wild-type tumors, *p* = 0.69, log-rank test) [4]. To date, the prognostic impact of *BRCA*1 expression in HGSOC without *BRCA*1 mutations is still unclear. This alteration has not been shown to be predictive of long responses to PARPi, and this is currently being tested in other cancers [28].

#### 2.1.3. Mutations in HR Genes in BRCA1/2 Wild-Type Patients

As stated previously, *BRCA*1/2 defects are only present in a small portion of patients with HGSOC. Whether other HR-related genetic alterations present the BRCAness phenotype and response to PARPi is partly unknown. Kang et al. developed a score based on the expression of 23 genes related to DNA-repair mechanisms and using data from 511 patients studied in the TCGA-Ov. These 23 genes were selected based on a previous literature review and knowledge of the DNA-repair pathways of the authors. The group of patients with high scores (high expression) had increased five-year OS (40% vs. 17% in the low-score group). This score proved to be a more reliable prognostic factor than classical clinical ones in the receiver operating characteristic (ROC) curves (area under the curve (AUC): 0.65 vs. 0.52), and was correlated with response rates and PFS after the first line with platinum [34]. Subsequently, Pennington et al. showed similar prognoses and response rates to platinum salts between germline *BRCA*1/2-mutated tumors and those with mutations in *ATM*, *BARD1*, *BRIP1*, *CHEK1*, *CHEK2*, *FAM175A*, *MRE11A*, *NBN*, *PALB2*, *RAD51C*, and *RAD51D* in a retrospective study of 390 samples of which 31% harbored one of these alterations [35]. These genes have been related to DHR through assays in-vitro [36,37]. Preliminar clinical data of PARPi efficacy in these patients come from ARIEL3 trial. In this study, mutational status of these and other 17 HR-related genes (apart from *BRCA*1/2) was used for stratification. Forty-three patients harboring mutations in these genes were identified and showed particular sensitivity to rucaparib (28 in the rucaparib arm/15 in the placebo arm). The value of these defects as predictive factors of response to olaparib is being investigated in the ORZORA trial (NCT02476968).

#### 2.1.4. Detecting “Genomic Scars”

Another strategy for the identification of tumors with DHR is to detect unique patterns of DNA damage and repair, the so-called “genomic scar”. Several genomic methods have been investigated for this purpose, but those based on SNP assays are the most developed [28]. Their objective is to quantify facts such as allele telomeric imbalance, the percentage of genome-wide LOH and large-scale transitions, which are hints of genomic abnormalities derived from defects in DNA-repair mechanisms [38]. The use of any of these scales individually or in combination has led to the development of different molecular tests to determine the state of HR (deficient or competent). These tests are performed on paraffin-embedded tissue and have already been used in clinical trials with PARPi in ovarian cancer. The My Choice test (Myriads) used in the NOVA study combines these three scales and its result is predictive of the response to N in terms of PFS, although all subgroups of patients largely benefited from this drug, as stated in the Introduction of this article (hazard ratios ranging from 0.27 in germline *BRCA*1/2 mutated patients to 0.58 in *BRCA*1/2 wild-type HR proficient patients, the latest being an exploratory analysis). The ARIEL2 trial, a phase II trial assessing R sensitivity in prospectively defined molecular groups, presented the LOH (using a cutoff = 14%) as a potential biomarker of the response to this drug in patients with platinum-sensitive relapse after one or more prior platinum chemotherapy lines [26]. However, ARIEL3 failed to validate LOH (cutoff = 14%) as a predictive biomarker of sensitivity to R in the maintenance setting after platinum for relapses, showing a hr of 0.44 in the subgroup of *BRCA*1/2 wild-type patients with high levels of LOH versus 0.58 in the subgroup of *BRCA*1/2 wild-type patients with low levels, both being preplanned analyses. On the whole, the results of trials that evaluated N and R in a maintenance setting showed that these tests are not able to efficiently discriminate between patients who may obtain a significant benefit and those who may not [39].

#### 2.1.5. Determining HR Real Status

However, the detection of “genomic scars” reflect cellular events that occurred in the past, rather than the current status of HR. Because DHR can be reversible (i.e., when secondary *BRCA*1/2 mutations appear) [28], several groups are investigating the development of functional tests based on the quantification of RAD51 foci in response to DNA damage by means of immunohistochemistry or immunofluorescence- its absence is a feature of DHR. However, the requirement of fresh tissue makes their use in daily practice difficult, and there are not available techniques yet [40,41,42,43].

The results of the ARIEL3 and NOVA trials, showing that all patients with HGSOC benefit from PARPi as a maintenance treatment (to a greater or lesser degree), cast doubt on the need for the aforementioned tests. There are no clear biological explanations for these results, but it is important to remark that PARPs are essential enzymes in several cellular functions, some of which are only partially known [11,44]. However, the determination of HR status could be used to optimize future therapeutic armamentarium. Learning the biological mechanism of action of PARPi in tumors with competent HR will contribute to the development of new strategies in this group of patients. In this sense, very recently, Zimmermann et al. reported their preclinical findings in cell lines in which clustered regularly interspersed palindromic repeats (CRISPR) technology identified that mutations in the three genes encoding ribonuclease H2, and thus impaired ribonucleotide excision repair, predicted *in-vitro* hypersensitivity to PARPi [45].

### 2.2. In Which Setting Should PARPi Be Used?

As stated before, PARPi have been approved in different settings by the FDA and EMA [16,18]. Very briefly, maintenance approvals are focused on patients with response to platinum used for relapse, while treatment approvals are focused on pretreated patients with deleterious *BRCA*1/2 mutated epithelial ovarian cancer, both for platinum-resistant or sensitive relapses. In summary, data from large phase III trials have provided strong evidence for the maintenance setting, but the use of PARPi as a treatment for relapse is based on phase II trials with fewer than 200 patients each. Currently, results from large trials assessing the role of R, O and N as treatment at relapse are awaited:

- The ARIEL4 trial (NCT02855944), a phase III currently under accrual, aims to compare rucaparib to chemotherapy as a treatment of ovarian cancer relapses in *BRCA*1/2-mutant patients, excluding only platinum-refractory patients.
- Olaparib is also being studied in two phase III trials as treatment for platinum-sensitive relapses (results pending): in SOLO3, O is compared to non-platinum chemotherapy in germline *BRCA*1/2-mutated patients who have received at least two prior platinum treatments (NCT02282020), and in GY004, O is being compared to cediranib plus O and standard platinum-based chemotherapy (3 arms in total) (NCT02446600).
- Final results of QUADRA (a large phase II with 500 participants), exploring niraparib as a treatment at relapse in highly pretreated patients, are awaited (NCT02354586) [29].

In summary, the optimal setting is still unknown. Clone selection after chemotherapy is a key question to be considered, since the use of PARPi as a maintenance therapy after response to platinum agents or as a treatment for relapses target different population of cells.

On the other hand, PARPi use as maintenance immediately after the first chemotherapy line is currently being investigated in large randomized trials. Final published results are awaited from the SOLO1 trial (NCT01844986), which has tested O in germline *BRCA*1/2-mutated patients. Noticeably, a very recent press release from AstraZeneca in June 2018 communicated a significant improvement in PFS (SOLO1 press release 27 June 2018, www.astrazeneca.com). Also, results from the PAOLA1, a phase III trial testing maintenance with O added to the standard regimen carboplatin/paclitaxel/bevacizumab in “all-comers”, are pending (NCT02477644). N has been tested in the PRIMA trial as a maintenance drug after first line chemotherapy (results pending, NCT02655016). Finally, veliparib (PARPi still in clinical development) is being investigated in a large phase III trial comparing three arms: carboplatin/paclitaxel versus carboplatin/paclitaxel/veliparib versus carboplatin/paclitaxel/veliparib followed by veliparib as maintenance (results pending, NCT02470585) [29]. Therefore, several clinical trial results are pending, but based on the close relationship between platinum-sensitivity and PARPi sensitivity, it can be hypothesized that using PARPi at earlier stages of the disease may increase their efficacy and the number of patients who benefit from them.

### 2.3. Trying to Overcome Resistance to PARPi

Despite the initial and sometimes prolonged response to PARPi, most patients with HGSOC will eventually develop resistance to them. The study of the mechanisms of resistance to these drugs can provide key knowledge to guide their future clinical development and improve their clinical results. These mechanisms can be conceptually divided into those that restore HR and those that do not. Only some of them have been identified in the clinic [46].

Among HR-restorative mechanisms, the most featured one are secondary *BRCA*1/2 mutations. Much clinical evidence shows the presence of secondary mutations that functionally restore BRCA1 and BRCA2 proteins in platinum-resistant ovarian tumors [46,47,48], and also in *BRCA*1/2-mutated ovarian carcinomas that are resistant to olaparib [7,49]. In a cohort of 26 platinum-resistant ovarian cancer patients carrying *BRCA*1/2 mutations, 46.2% had secondary mutations [50]. Recently, secondary mutations in *RAD51C* and *RAD51D* were reported in six patients with rucaparib-resistant ovarian cancer [51]. However, the frequency of these events in patients treated with PARPi is unknown.

Other HR-restorative mechanisms only described in preclinical work affect the imbalance between HR and NHEJ. Preclinical evidence supports the loss of p53 (P53BP1) expression and the consequent NHEJ impairment as a mechanism of resistance to PARPi in *BRCA*1-deficient cell lines [46]. The P53BP1 is a mediator of the NHEJ, which is a DNA damage-repair system that is activated alternatively to HR through fine cellular regulation depending on RAP80, among others [52]. Bouwman et al. showed that P53BP1 is essential for sustaining growth arrest induced by deficient BRCA1, given that its absence allows for the recruitment of RAD51, even in BRCA1-deficient cells, and it can thus restore HR, according to observations in murine models [53,54]. In addition, its dysfunctional mutated status has been identified in *BRCA*1-mutated, PARPi-resistant, murine breast-cancer models [55]. PARPi resistance related to loss of P53BP1 may be enhanced by mutant BRCA1 stabilization secondary to heat shock protein 90 (HSP90) [56]. HR can also be restored by the deficiency of other factors that promote NHEJ, such as *JMJD1C* [57], *REV*7 [58,59], or *RIF1* [60], or the overexpression of microRNA622 [61].

On the other hand, a decreased expression of PARP enzymes [46], the overexpression of *FANCD2* [62] or *SLFN11* inactivation [63] have been postulated as potential not HR-restoring mechanisms of resistance to PARPi. These and other events have been related to PARPi resistance in the preclinical setting but clinical validation has not been performed yet. The relationship of these alterations to platinum resistance has not been well-described to date [7,64].

Regarding PARPi pharmacology, olaparib resistance mediated by the overexpression of transporter protein genes (such as the transmembrane pump PgP or ABCB1) has been described in murine models of breast cancer associated with *BRCA*1 mutations [7]. In a previous study, 8% of relapsed HGSOC samples overexpressed *ABCB1* [50]. These mechanisms are potentially reversible with the coadministration of PgP inhibitors and migh not be common to other PARPi.

The impact of the above-described mechanisms of resistance to PARPi, in terms of frequency in a clinical setting, is unknown. Whether they are drug-dependent or class-dependent, and their relevance according to basal patient characteristics (proficient or deficient HR, for instance) are also unknown in most cases. Basic and clinical research in this field should provide key information to increase PARPi efficacy and to guide therapeutic management upon progression to PARPi. At present, the usefulness of intermittent (on/off) strategies or the sequential use of different PARPi are still undetermined. Nowadays, clinical research is mainly orientated to potential combinations including PARPi [65], as will be detailed in the next section.

Finally, some of the presented data questions the value of the platinum-free interval to determine whether a new treatment with platinum is appropriate after progression to PARPi, as some of the mentioned mechanisms of resistance may also explain platinum-resistance. However, if we look at the clinical data from the SOLO2 trial, there is also significant benefit in the time to second subsequent therapy (median TSST in the olaparib arm not reached versus 18 months in the placebo arm) [22]. Moreover, rechallenge with PARPi is currently under clinical investigation, either as a maintenance option after subsequent platinum retreatment (such as the case of OREO trial with olaparib, NCT03106987) or in combination with other drugs after progression on olaparib (such as olaparib with cediranib, NCT02340611) [29].

### 2.4. Potential Drug Combinations Including PARPi for Ovarian Cancer Patients

With the aim of increasing PARPi efficacy and overcoming their resistance, O, N, R, veliparib, and talazoparib (another PARPi still under clinical development), are involved in several combination trials. Main strategies and some relevant results are described in this section. Recruiting trials exploring different combinations with PARPi in ovarian cancer patients (and a representation of those active trials with results pending) are detailed in Table 2 [29].

#### 2.4.1. Combinations with Chemotherapy

Some chemotherapy agents are potential companions to PARPi due to their ability of inducing DNA damage, and this area is being increasingly studied (Reviewed in Matulonis 2017 [66]). Selection of specific drugs can potentiate inhibition of PARP catalytic activity and/or PARP trapping, and specific combinations may act synergistically or additively. Moreover, overlapping myelosupression may be a dose limiting toxicity [66].

Combinations with DNA damaging anticancer agents such as platinum compounds or alkylating agents have been specifically assessed in ovarian cancer. Regarding platinum compounds, two separate trials assessing the feasibility of olaparib or veliparib in combination with carboplatin/paclitaxel have been reported, with a metronomic and a standard regimen, respectively [67,68]. Additionally, in a randomized phase II trial, olaparib plus carboplatin (AUC 4)/paclitaxel followed by olaparib as maintenance significantly improved PFS versus the chemotherapy doublet alone (AUC 6 for carboplatin), with a hr of 0.51 in the intention-to-treat population analysis (*n* = 173, 12.2 vs. 9.6 months), and had an acceptable and manageable tolerability profile. A prespecified exploratory analyses of *BRCA*1/2-mutated patients (retrospectively assessed) showed a hr of 0.21 in the germline *BRCA*1/2-mutated patients, *n* = 41) [69]. The authors explained that one of their aims was to explore the extent by which the addition of olaparib potentiates the chemotherapy cytotoxic effect. Taking into account the small differences in response rates between the two arms and the late separation of the PFS curves, they concluded that an additive effect to the lower carboplatin dose may be suggested, and that the maintenance phase was probably the key contributor to the observed clinical benefit of the combination. Development of O, N and R as maintenance therapies has not included immediately prior combination with chemotherapy (during the treatment phase). Nontheless, as stated before, results from a large phase III trial testing the addition of veliparib both in the chemotherapy phase and the maintenance phase are awaited (NCT02470585). However, important myelosupressive toxicity and hypertension were observed with the combination of carboplatin/pegilated liposomal doxorubicin/bevacizumab/veliparib in a phase I trial [70]. New strategies such as intermittent administration of PARPi concurrently to chemotherapy are being current studied [71].

Regarding alkylating agents, the addition of veliparib has not proved to provide any benefit to cyclophosphamide when treating platinum-resistant relapsed ovarian cancers in germline *BRCA*1/2 mutated patients [72], and results are not available from its combination with temozolamide (NCT00526617, NCT01113957).

On the other hand, some preclinical studies suggest a synergistic effect between PARPi and topoisomerase I inhibitors, due to enhanced inhibition of both enzymes [73]. In this sense, published results of a phase I testing the combination of veliparib and irinotecan showed acceptable tolerability and 19% of responses, correlating with specific changes in the performed pharmacodynamics studies [74].

Also combinations of PARPi with topoisomerase II inhibitors (liposomal doxorubicin) and cytotoxic agents with different mechanism of action are currently being tested (mirvetuximab soravtansine or lurbinectidine, see Table 2).

#### 2.4.2. Combinations with Selective DNA Damage-Repair Inhibitors

Combination of PARPi with targeted agents that negatively influence HR could overcome HR-restoration and enhance PARPi efficacy in HR proficient tumors. The underlying rationale for these combinations is again the concept of synthetic lethality, this time chemically induced: by concurrently blocking alternative DNA damage-repair pathways, cancer cells become unviable [75,76]. This strategy could therefore sensitize primary or acquired (upon restoration) HR proficient tumors to PARPi. Currently studied companions are inhibitors of HSP90 (onalespib), WEE1 (Adavosertib), ATM/ATR (AZD6738), and antiangiogenic agents (cediranib, bevacizumab) (see Table 2).

The ATM-CHK2 pathway and the ATR/CHK1/WEE1 pathway have a key role in cell-cycle regulation. They are targets of cell-cycle checkpoints inhibitors, which abrogate S and G2 arrests and therefore impair normal DNA-damage repair before mitosis is completed [77]. Clinical results from their combinations with PARPi are awaited. 

Hypoxia induced by antiangiogenic agents seem to downregulate *BRCA*1/2 and *RAD51* in cancer cells [78,79]. Remarkably, cediranib (a VEGFR3 inhibitor) has already shown very positive results in combination with olaparib in a phase II trial with 90 patients with recurrent platinum-sensitive HGSOC tumors, particularly in those *BRCA*1/2 wild-type. This combination showed 17.7 months in PFS compared to 9 months with olaparib alone in the intention-to-treat population, while a post-hoc exploratory analyses showed 16.5 and 5.7 months, respectively, in *BRCA*1/2 wild-type patients [80]. This result has led to a plethora of trials assessing different combinations of a PARPi and an antiangiogenic agent.

Other potential druggable targets are RAD51 [81,82], RAD52 [83,84] and proteins involved in DNA-damage repair pathways other than HR, such as polymerase-θ (Polθ) involved in microhomology-mediated end joining (MMEJ, an error-prone DDR pathway hyperactivated in DHR cells) [85,86].

#### 2.4.3. Combinations with PI3K Pathway Inhibitors

Combinations with inhibitors of PI3K or mTORC1/2 are also being investigated (see Table 2). The rationale for this strategy is the observed preclinical synergistic activity of this combination in murine models of breast cancer (either *BRCA*1-related or sporadic triple-negative) [87]. Taking into account that up to 45% of HGSOC patients present deregulation of this pathway [4], this strategy is of high interest. Published results of a phase I trial combining BKM120 and olaparib reported grade 3 depression, transaminitis and hyperglycemia as DLTs of BKM120, though the combination was feasible [88]. Recommended phase II dose of AZ5363, an AKT inhibitor, for combining with olaparib has been recently communicated [89]. Currently ongoing trials assessing these combinations are detailed in Table 2.

#### 2.4.4. Combinations with Immunotherapies

The increased number of neoantigens released after PARPi-induced tumor cell apoptosis could facilitate immunoresponse against the tumor. Moreover, immunomodulatory effects of PARPi have been observed in vitro [90]. This serves as rationale for testing combinations of PARPi and immunotherapies. Of note, preliminary results from the recurrent ovarian cancer cohort included in TOPACIO/Keynote-162 (NCT02657889) trial were recently reported in ASCO. This phase 1/2 study of niraparib + pembrolizumab showed 25% ORR in the 39 platinum-resistant patients and 45% of objective response rates in *BRCA*1/2-mutant patients [91]. Several different combinations are under accrual (see Table 2).

## 3. Conclusions

DHR is present in approximately 50% of HGSOC. Patients with these tumors exhibit better prognose than those with competent HR, as well as prolonged responses to PARPi, due to the mechanism of action of these drugs. Therefore, DHR constitutes a therapeutic opportunity thanks to PARPi. Although initially, these drugs were developed for patients with *BRCA*1/2 mutations, robust clinical data showing their benefit in a broader population without DHR are now available. 

This breakthrough in daily practice has raised several clinical questions: How can populations that will most benefit from PARPi be selected? At which stage of ovarian cancer should PARPi be used? What clinical strategies could overcome resistance to PARPi? Which strategies are reasonable after progression to PARPi? 

From the authors’ perspective, this scenario represents a great opportunity for translational research. On the one hand, learning the impact of specific *BRCA*1 or *BRCA*2 mutations and developing functional tests of HR status will help to define the most PARPi sensitive population. Secondly, featuring differences between cell clones present at relapse and those selected under chemotherapy pressure may impact on a differential clinical development for the treatment or maintenance setting. 

The study of the mechanisms of resistance to PARPi is also highly interesting. Learning PARPis’ mechanism of action in HR proficient tumors (a fact already proven in phase III trials) while acknowledging that HR-restoration is a known mechanism of resistance is an intriguing question. The frequency of the above-described mechanisms of resistance to PARPi in a clinical setting is unknown. Outstandingly, the field of potential combinations is currently under extensive clinical development. Translational research on the underlying mechanisms of action of such combinations is of high importance. 

In conclusion, this is a field in which quick clinical drug development and translational research may act synergistically to improve strategies for disease control and eventually patients’ outcomes.

## Figures and Tables

**Table 1:** History of PARPi approvals in ovarian cancer.

	OLAPARIB	NIRAPARIB	RUCAPARIB
EMA	**Jan 2015:** —Maintenance treatment of patients with platinum-sensitive relapsed BRCA-mutated (germline and/or somatic) HGSOC who are in response to platinum-based chemotherapy **Feb 2018:** positive opinion on the extension of marketing authorization of olaparib tablets for patients regardless of the presence of *BRCA*1/2 mutations.	**Nov 2017:** —Maintenance treatment of patients with platinum-sensitive relapsed HGSOC who are in response to platinum-based chemotherapy	**May 2018:** —Treatment of adult patients with platinum sensitive, relapsed or progressive, BRCA mutated (germline and/or somatic) HGSOC, who have been treated with two or more prior lines of platinum based chemotherapy, and who are unable to tolerate further platinum based chemotherapy
FDA	**Dec 2014:** —Treatment after 3 lines of chemotherapy for relapse, in germline BRCA mutated advanced ovarian cancer **Aug 2017:** —Maintenance treatment of patients with recurrent epithelial ovarian cancer, who are in response to platinum-based chemotherapy.	**Oct 2016:** —Maintenance treatment of patients with platinum-sensitive relapsed HGSOC who are in response to platinum-based chemotherapy	**Dec 2016:** —Treatment of patients with deleterious BRCA mutation (germline and/or somatic) associated advanced ovarian cancer who have been treated with two or more chemotherapies **Apr 2018:** —Maintenance treatment of recurrent epithelial ovarian cancer who are in response to platinum-based chemotherapy

**Table 2:** Current recruiting trials combining PARPi with other drugs in ovarian cancer (and some examples of active trials not recruiting with pending results).

Combinational Drug	PARPi	NCT	Title	Trial Status
Carboplatin and Paclitaxel	Veliparib	NCT02470585	Veliparib With Carboplatin and Paclitaxel and as Continuation Maintenance Therapy in Subjects with Newly Diagnosed Stage III or IV, High-grade Serous, Epithelial Ovarian, Fallopian Tube, or Primary Peritoneal Cancer (phase III)	Active, not recruiting (pending results)
Mirvetuximab Soravtansine	Rucaparib	NCT03552471	Mirvetuximab Soravtansine and Rucaparib Camsylate in Treating Participants with Recurrent Endometrial, Ovarian, Fallopian Tube or Primary Peritoneal Cancer.	Recruiting
Lurbinectidine	Olaparib	NCT02684318	Study to Evaluate PM01183 in Combination with Olaparib in Advanced Solid Tumors.	Recruiting
Liposomal Doxorubicin	Olaparib	NCT03161132	Resistant Ovarian Cancer, Olaparib and Liposomal Doxorubicin (ROLANDO).	Recruiting
Floxuridine	Veliparib	NCT01749397	Veliparib and Floxuridine in Treating Patients with Metastatic Epithelial Ovarian, Primary Peritoneal Cavity, or Fallopian Tube Cancer.	Active, not recruiting (pending results)
Onalespib	Olaparib	NCT02898207	Olaparib and Onalespib in Treating Patients with Solid Tumors That Are Metastatic or Cannot Be Removed by Surgery or Recurrent Ovarian, Fallopian Tube, Primary Peritoneal, or Triple-Negative Breast Cancer.	Recruiting
AZD6738	Olaparib	NCT03462342	Combination ATR and PARP Inhibitor (CAPRI) Trial with AZD6738 and Olaparib in Recurrent Ovarian Cancer.	Recruiting
Adavosertib	Olaparib	NCT03579316	Adavosertib With or Without Olaparib in Treating Participants with Recurrent Ovarian, Primary Peritoneal, or Fallopian Tube Cancer.	Not yet recruiting
Bevacizumab	Niraparib	NCT02354131	Niraparib Versus Niraparib-bevacizumab Combination in Women with Platinum-sensitive Epithelial Ovarian Cancer.	Accrual completed (Part2 pending results)
Bevacizumab	Niraparib	NCT03326193	Phase 2, A Study of Niraparib Combined with Bevacizumab Maintenance Treatment in Patients with Advanced Ovarian Cancer Following Response on Front-Line Platinum-Based Chemotherapy.	Recruiting
Bevacizumab	Rucaparib	NCT03462212	Trial of Carboplatin-Paclitaxel-Bevacizumab vs Carboplatin-Paclitaxel-Bevacizumab-Rucaparib vs Carboplatin-Paclitaxel-Rucaparib in Patients with Advanced (Stage III B-C-IV) Ovarian, Primary Peritoneal and Fallopian Tube Cancer. (MITO25) (NOTE: rucaparib only during the maintenance phase).	Recruiting
Cediranib	Olaparib	NCT02889900	Efficacy and Safety Study of Cediranib in Combination with Olaparib in Patients with Recurrent Platinum-Resistant Ovarian Cancer.	Recruiting
Cediranib	Olaparib	NCT02340611	A Study of Cediranib and Olaparib at the Time Ovarian Cancer Worsens on Olaparib.	Completed accrual (pending results)
Cediranib	Olaparib	NCT03278717	Study Evaluating the Efficacy of Maintenance Olaparib and Cediranib or Olaparib Alone in Ovarian Cancer Patients.	Not yet recruiting
Cediranib	Olaparib	NCT02681237	A Study of Cediranib and Olaparib at Disease Worsening in Ovarian Cancer.	Recruiting
Cediranib	Olaparib	NCT03117933	Olaparib +/− Cediranib or Chemotherapy in Patients with BRCA Mutated Platinum-resistant Ovarian Cancer.	Recruiting
Cediranib	Olaparib	NCT03314740	Best Approach in Recurrent-Ovarian-Cancer-with Cediranib-Olaparib (BAROCCO).	Recruiting
Cediranib	Olaparib	NCT02446600	Olaparib or Cediranib Maleate and Olaparib Compared with Standard Platinum-Based Chemotherapy in Treating Patients with Recurrent Platinum-Sensitive Ovarian, Fallopian Tube, or Primary Peritoneal Cancer (phase III).	Active, not recruiting (pending results)
Everolimus	Niraparib	NCT03154281	Evaluation of the Safety and Tolerability of Niraparib With Everolimus in Ovarian and Breast.	Recruiting
Copanlisib	Niraparib	NCT03586661	Niraparib and Copanlisib in Treating Participants with Recurrent Endometrial, Ovarian, Primary Peritoneal, or Fallopian Tube Cancer.	Recruiting
Buparlisib or Alpelisib	Olaparib	NCT01623349	Phase I Study of the Oral PI3kinase Inhibitor BKM120 or BYL719 and the Oral PARP Inhibitor Olaparib in Patients with Recurrent Triple Negative Breast Cancer or High Grade Serous Ovarian Cancer.	Active, not recruiting (partially pending results)
Vistusertib or AZD5363	Olaparib	NCT02208375	A Phase Ib Study of the Oral PARP Inhibitor Olaparib With the Oral mTORC1/2 Inhibitor AZD2014 or the Oral AKT Inhibitor AZD5363 for Recurrent Endometrial, Triple Negative Breast, and Ovarian, Primary Peritoneal, or Fallopian Tube Cancer.	Active, not recruiting (pending results)
TSR-042	Niraparib	NCT03602859	A Phase 3 Comparison of Platinum-Based Therapy With TSR-042 and Niraparib Versus Standard of Care Platinum-Based Therapy as First-Line Treatment of Stage III or IV Nonmucinous Epithelial Ovarian Cancer.	Not yet recruiting
Atezolizumab	Niraparib	NCT03598270	Platinum-based Chemotherapy with Atezolizumab and Niraparib in Patients with Recurrent Ovarian Cancer (ANITA).	Recruiting
Pembrolizumab	Niraparib	NCT02657889	Niraparib in Combination with Pembrolizumab in Patients with Triple-negative Breast Cancer or Ovarian Cancer (TOPACIO).	Active, not recruiting (partially pending results)
Nivolumab	Rucaparib	NCT03522246	A Study in Ovarian Cancer Patients Evaluating Rucaparib and Nivolumab as Maintenance Treatment Following Response to Front-Line Platinum-Based Chemotherapy (ATHENA).	Recruiting
Avelumab	Talazoparib	NCT03642132	Avelumab and Talazoparib in Untreated Advanced Ovarian Cancer (JAVELIN OVARIAN PARP 100).	Recruiting
Durvalumab & Tremelimumab	Olaparib	NCT02953457	Olaparib, Durvalumab, and Tremelimumab in Treating Patients with Recurrent or Refractory Ovarian, Fallopian Tube or Primary Peritoneal Cancer with BRCA1 or BRCA2 Mutation.	Recruiting
Tremelimumab	Olaparib	NCT02571725	PARP-inhibition and CTLA-4 Blockade in BRCA-deficient Ovarian Cancer.	Recruiting
MEDI4736	Olaparib	NCT02734004	A Phase I/II Study of MEDI4736 in Combination with Olaparib in Patients with Advanced Solid Tumors.	Recruiting
MEDI4736 cediranib	Olaparib	NCT02484404	Phase I/II Study of the Anti-Programmed Death Ligand-1 Antibody MEDI4736 in Combination with Olaparib and/or Cediranib for Advanced Solid Tumors and Advanced or Recurrent Ovarian, Triple Negative Breast, Lung, Prostate and Colorectal Cancers.	Recruiting
Tsr-042 Bevacizumab	Niraparib	NCT03574779	Phase 2 Multicohort Study to Evaluate the Safety and Efficacy of Novel Treatment Combinations in Patients with Recurrent Ovarian Cancer (OPAL): tsr42, BEVA.	Not yet recruiting
INCB057643	Rucaparib	NCT02711137	Open-Label Safety and Tolerability Study of INCB057643 in Subjects with Advanced Malignancies.	Active, not recruiting
Selumetinib	Olaparib	NCT03162627	Selumetinib and Olaparib in Solid Tumors.	Recruiting
